# Comprehensive transcriptional profiling of NaCl-stressed Arabidopsis roots reveals novel classes of responsive genes

**DOI:** 10.1186/1471-2229-6-25

**Published:** 2006-10-12

**Authors:** Yuanqing Jiang, Michael K Deyholos

**Affiliations:** 1 Department of Biological Sciences, University of Alberta, Edmonton, Canada

## Abstract

**Background:**

Roots are an attractive system for genomic and post-genomic studies of NaCl responses, due to their primary importance to agriculture, and because of their relative structural and biochemical simplicity. Excellent genomic resources have been established for the study of Arabidopsis roots, however, a comprehensive microarray analysis of the root transcriptome following NaCl exposure is required to further understand plant responses to abiotic stress and facilitate future, systems-based analyses of the underlying regulatory networks.

**Results:**

We used microarrays of 70-mer oligonucleotide probes representing 23,686 Arabidopsis genes to identify root transcripts that changed in relative abundance following 6 h, 24 h, or 48 h of hydroponic exposure to 150 mM NaCl. Enrichment analysis identified groups of structurally or functionally related genes whose members were statistically over-represented among up- or down-regulated transcripts. Our results are consistent with generally observed stress response themes, and highlight potentially important roles for underappreciated gene families, including: several groups of transporters (e.g. MATE, LeOPT1-like); signalling molecules (e.g. PERK kinases, MLO-like receptors), carbohydrate active enzymes (e.g. XTH18), transcription factors (e.g. members of ZIM, WRKY, NAC), and other proteins (e.g. 4CL-like, COMT-like, LOB-Class 1). We verified the NaCl-inducible expression of selected transcription factors and other genes by qRT-PCR.

**Conclusion:**

Micorarray profiling of NaCl-treated Arabidopsis roots revealed dynamic changes in transcript abundance for at least 20% of the genome, including hundreds of transcription factors, kinases/phosphatases, hormone-related genes, and effectors of homeostasis, all of which highlight the complexity of this stress response. Our identification of these transcriptional responses, and groups of evolutionarily related genes with either similar or divergent transcriptional responses to stress, will facilitate mapping of regulatory networks and extend our ability to improve salt tolerance in plants.

## Background

Roots are the primary site of perception and injury for several types of water-limiting stress, including salinity and drought. In many circumstances, it is the stress-sensitivity of the root that limits the productivity of the entire plant [1,2]. The physiological significance of roots is belied by their relative structural simplicity as compared to other plant organs: roots are largely lacking in some major metabolic pathways such as photosynthesis, and have a stereotypical morphology that is conserved across taxa and throughout the life cycle of individuals. This combination of physiological relevance and structural simplicity has made roots obvious targets for functional genomic analyses. For example, detailed transcriptional profiles have now been resolved to single cell types within roots, and these are now being integrated into regulatory circuits and networks [3].

Salinity treatments of plants are also an attractive experimental system. High salinity (generally meaning NaCl accumulation in soil) is estimated to reduce agricultural productivity on more than 20% of the world's cultivated land [4]. NaCl treatments are simple to apply in laboratory settings, and dosage and timing can be controlled more precisely than with other major abiotic stresses such as chilling, freezing, and dehydration. Accordingly, microarray-based analyses of the response of Arabidopsis to NaCl have been published in at least nine reports. However, most of these studies have analyzed either cell cultures or whole plants, rather than specific tissues [5-10]. Of the previous studies that analyzed roots specifically, none used microarray probe sets representing more than 8,100 of the originally predicted 25,498 genes in the Arabidopsis genome [11-14]. Although Affymetrix microarrays containing probes for at least 22,591 Arabidopsis genes have been used to profile NaCl responses specifically in roots, these data were generously deposited to public databases, but without detailed description or analysis in the primary literature [15]. Thus, the absence of available, comprehensive transcriptomic data describing the response of Arabidopsis roots to NaCl treatment, in combination with the potential applications of these data in molecular physiology and systems biology, motivated us to conduct the research we describe here.

## Results and discussion

### Whole-plant responses to salt treatment

**Figure 1:**
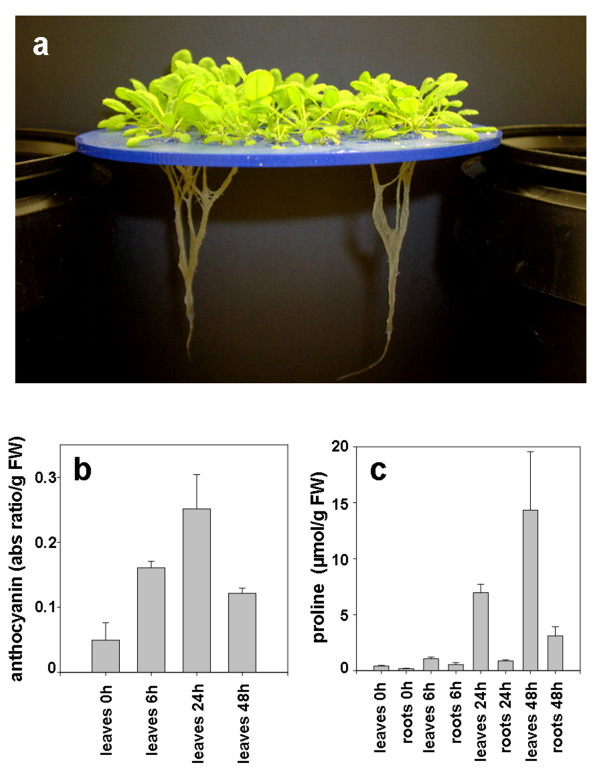
**Hydroponic growth system and physiological responses to NaCl treatments. **a) A raft of Arabidopsis plants at 21 dpi, preceding treatment. This raft has been removed from the hydroponic growth medium for the purpose of this photograph; although individual roots cling together in air, they are well separated when submerged in the growth medium. b) Anthocyanin accumulation in Arabidopsis leaves. Plants were mock treated or exposed to 150 mM NaCl for 6, 24 and 48 h. The means of six experiments ± standard deviation are shown. Anthocyanin concentration is reported as an absorbance ratio: (A_530_-1/4*A_657_)/g fresh weight. c) Proline accumulation in roots and leaves of Arabidopsis plants. Arabidopsis plants were mock treated or exposed to 150 mM NaCl for 6, 24 and 48 h, and proline concentration was determined in both leaves and roots. The means of six experiments ± standard deviation are shown.

We applied a salt-shock treatment to 21 dpi (days past imbibition) Arabidopsis plants by supplementing their hydroponic growth medium with 150 mM NaCl (Figure 1). This concentration of NaCl has been used in several previous gene expression studies, since it induces a moderate stress response and is not acutely lethal [5,16]. Indeed, after application of 150 mM NaCl, we observed visible signs of stress including loss of turgor. However, even after 48 h of exposure to media supplemented with 150 mM NaCl, nearly all of the treated plants recovered and resumed growth when transferred into NaCl-free hydroponic medium (data not shown). Obviously, these experimental conditions differ from those experienced by soil-grown plants, especially field-grown crops, where multiple stresses and nutrient limitations can occur simultaneously [17]. Nevertheless, we expect that many stress response pathways will be conserved across treatments. This assumption, along with the precise experimental control afforded by laboratory hydroponics, justifies the use of hydroponics as model system for salinity stress.

To further define the physiological state of the plants that we subjected to transcriptional profiling, we monitored the accumulation of two stress-inducible metabolites, namely anthocyanin and proline [18]. Recent evidence suggests that anthocyanins may mitigate photooxidative injury by efficiently scavenging free radicals and reactive oxygen species [19]. Anthocyanin concentrations increased rapidly in leaves of stressed plants, with the absorbance of diagnostic wavelengths increasing nearly three-fold in the first 6 h after treatment (Figure 1). These pigments continued to accumulate, with a five-fold increase observed at 24 h after treatment. Interestingly, the concentration of anthocyanin began to decrease after 24 h of stress, due perhaps to catabolism of the accumulated pigment and acclimation to the stress conditions, resulting in levels of anthocyanin at 48 h post-stress that were slightly lower than levels observed at 6 h. Proline concentration increased rapidly in both the leaves and roots of NaCl-stressed plants until at least 48 h following NaCl treatment (Figure 1). We observed a 3-fold increase in proline concentration in roots after 6 h, and an 18-fold increase after 48 h. A similar pattern of increase, with higher absolute concentrations of proline, was also observed in shoots. The accumulation of these metabolites is an indication that these plants were actively mounting a stress response at the time we subjected them to transcriptional profiling.

### General transcriptomic responses

To characterize the effect of NaCl treatment on gene expression in Arabidopsis roots, we extracted RNA from control and stressed root samples at 6 h, 24 h, and 48 h following treatment, and analyzed these in paired, dye-reversed hybridizations on spotted 70-mer oligonucleotide microarrays consisting of probes representing 23,686 unique genes identified by Arabidopsis Genome Initiative (AGI) locus identifiers. The full data set has been deposited in the ArrayExpress database as accession E-MEXP-754 [20]. We identified probes for 7,217 unique genes whose treated:untreated log_2 _expression ratio differed significantly from 0 at one or more time points (please see Additional File 1), according to the Significance Analysis of Microarrays (SAM) algorithm, with a false discovery rate (FDR) of less than 5% [21]. Among these statistically significant expression ratios, probes representing 2,433 unique genes showed a NaCl-induced increase in transcript abundance of at least 2.0 fold at one or more time points. Conversely, probes representing 2,774 unique genes showed a decrease in abundance of at least 2.0 fold or more. Thus, transcript accumulation for at least 10% of Arabidopsis genes was strongly (i.e. > 2.0 fold) induced by NaCl-treatment, and transcripts for at least 12% of genes were equivalently repressed, in response to NaCl-treatment. The proportion of NaCl-responsive genes we observed is somewhat higher than previously reported estimates based on cDNA microarrays, likely reflecting the higher specificity of the oligonucleotide-based arrays [8].

### Validation of microarray results

We compared our results to publicly available microarray data from the AtGenExpress project, which used Affymetrix ATH1 arrays to describe the transcriptome of hydroponically grown Arabidopsis roots exposed to a 150 mM NaCl-shock for up to 24 h [15]. Qualitative trends in expression ratios are highly conserved between these datasets (Additional File 1). Quantitatively, we observed a Pearson correlation co-efficient of 0.77 (6 h) and 0.69 (24 h) when comparing the ATH1 expression values with our signal-intensity filtered ratios. The transcriptomic responses we describe here are therefore generally reproducible across platforms and between laboratories (see Additional File 1 for a comparison to data from other previous reports). Moreover, probes for at least 3,277 genes were deposited our spotted microarray that are not present on the ATH1 array, of which 730 were significantly responsive to NaCl treatment (Additional File 1). For example, the gene with the highest-fold induction (mean ratio = 7.4 (log_2_)) in our analysis is transcription factor *bHLH092 *(At5g43650), but this gene is not represented on the ATH1 array. Thus, although the Affymetrix ATH1 array and the Operon 1.0 70-mer probe set are each commonly described as "whole-genome", it is clear that either platform alone is deficient in the detection of hundreds of potentially significant genes, although in combination, they provide a nearly complete representation of the predicted transcriptome.

To provide further validation of our microarray data, we performed quantitative RT-PCR analysis on specific transcripts, including the highly inducible *bHLH092 *(Table 1). We selected 15 genes representing different functional categories, which according to our microarray analysis increased, decreased, or remained essentially unchanged in terms of transcript abundance following salt-shock. For all of these genes, the expression ratios measured by qRT-PCR and by microarray were highly correlated (r = 0.91, 0.92 and 0.88 for 6, 24 and 48 h treatment, respectively). These results help to confirm the general accuracy of the microarray data we present here. The functional significance of the NaCl-responsive genes we validated by qRT-PCR is discussed in more detail below.

### Classification of NaCl-responsive transcripts

**Figure 2:**
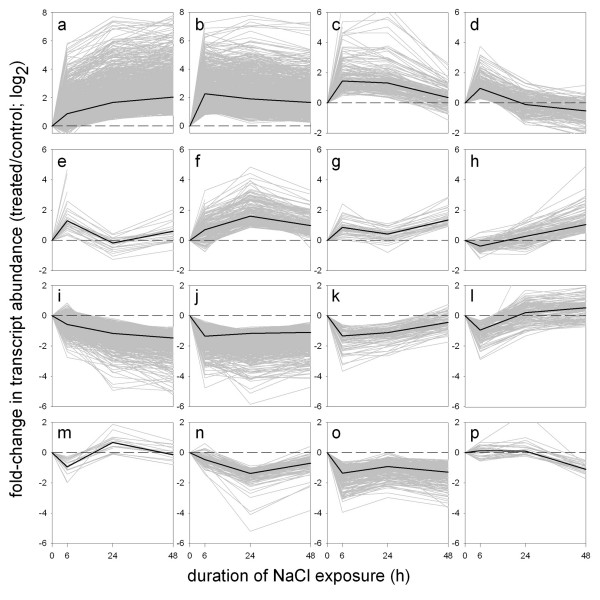
**NaCl-responsive transcripts grouped according to temporal expression profiles. **Microarray expression data that had been previously filtered for significance based on signal intensity and SAM [21] statistical analysis, was divided into 16 distinct temporal profiles, using STEM software [22]. Each of the profiles (labelled a-o) is represented as a different plot, with mean expression ratios (log_2_; treated:control) for each of the assigned transcripts at each time point plotted in grey. The median of all assigned expression ratios in each profile is plotted in black. To emphasize that the y-axis range is different between plots, a dashed line corresponding to a log_2_expression ratio of 0 is also shown in each plot.

We used the STEM (Short Time-series Expression Miner) software package to summarize our filtered microarray data, by clustering it into 16 distinct temporal expression patterns (Figure 2) [22]. The algorithms implemented in STEM are designed specifically for microarray experiments that sample only a few time points, such as ours. STEM accordingly minimizes the potential for over-fitting that can occur when some other clustering methods are applied to time course data, and also facilitates the identification of over-represented functional categories within each cluster, as described below.

The predominant temporal expression patterns detected by STEM analysis show that changes in transcript abundance can be detected within the first six hours following treatment for the majority of the 5,463 NaCl-responsive genes represented in the profiles (Figure 2.). At subsequent time points (i.e. 24 h, 48 h) the response generally either continued along the same trajectory (Figure 2a,i), or remained close to the level of induction or repression observed at 6 h (Figure 2b,j). Another subgroup of genes had a peak response at 6 h, which returned towards untreated levels of transcript abundance either gradually (Figure 2c,k), or more rapidly (Figure 2d,e,l,m). It is notable that a significant number of these genes show opposite patterns of induction and repression at 6 h as compared to 48 h (Figure 2d,l). Still other genes that were responsive at 6 h showed a peak change in expression at 24 h (Figure 2f,n), or, surprisingly, a moderate dampening of the response at 24 h, and a renewed intensity of response at 48 h (Figure 2g,o). Finally, in contrast to all of the patterns discussed so far, which began with a marked response at 6 h, a limited but significant number of genes showed relatively little response until the 48 h time point (Figure 2h,p). Thus, although the majority of genes are responsive by 6 h of treatment, dynamic patterns of gene expression are also detected after 24 h and 48 h of treatment. These observations indicate that different regulatory networks and effectors are active at each of these different periods following the perception of stress.

As an objective means of generalizing the biological functions represented by NaCl-responsive transcripts, we next used STEM to identify categories of functionally and/or structurally related genes that are enriched (i.e. statistically over-represented at FDR < 5%) in one or more of the clusters described above. Preliminary analysis using Gene Ontology consortium categories (data not shown) was used to guide the selection of a detailed list of gene families and superfamilies from primary literature sources, as well as from curated databases including KEGG (Kyoto Encylcopedia of Genes and Genomes), AraCyc, and TAIR (The Arabidopsis Information Resource) [23-25]. The list of categories and corresponding genes can be found in Additional File 2. Most broadly, the functional themes represented by NaCl-responsive transcripts can be divided into effectors and regulators. According to this analysis, the most prominent effectors of the NaCl stress response are genes involved in detoxification, transport, protein turnover, energy metabolism, and production of osmoprotectants. The major classes of regulators detected are signal transduction components, transcription factors, and hormone related genes. Each of these broad themes has been previously reported in association with abiotic stress responses [26]. The analysis we present here highlights specific NaCl-responsive genes and groups of genes within these categories. Tables 2, 3, 4, 5, 6 show groups of functionally and/or phylogenetically related genes that, according to STEM analysis, were significantly over represented (FDR < 5%) in one or more STEM profile (Figure 2), as well as a limited number of other groups of genes mentioned in the text, for comparison. The tables also include the number of genes within each category that were up- or down-regulated more than 2-fold, although this 2-fold threshold is not a criterion *per se *in STEM analysis.

### Osmoprotectants

Osmoprotectants including proline and trehalose, and small hydrophilic proteins such as dehydrins help to maintain hydration of cellular components during osmotic stress [27]. Genes regulating levels of osmoprotectants are highly stress responsive and were among the first stress-inducible transcripts reported in the literature [28-31]. As expected, we observed induction of many of these genes in our NaCl-treated tissues (Table 2). Two proline biosynthetic genes were induced (*P5CR*, At5g14800; *P5CS*, At2g39800), consistent with the increase in proline abundance we observed when this metabolite was assayed directly (Figure 1). We also observed transcript-level induction of a large proportion of genes in the trehalose biosynthetic pathway including 7 of the 8 trehalose phosphatases detectable on our array, as well as some (but not all) dehydrins (Table 2). Although trehalose has been shown to act as an osmoprotectant in bacteria and fungi, recent studies have suggested that this disaccharide acts as a signalling molecule in plants, noting that its intracellular concentration is too low to be an effective osmoprotectant [32].

### Reactive oxygen network

NaCl treatment, like many environmental stresses, generates reactive oxygen species (ROS)[33]. These ROS and the products of their reactions with other cellular components (e.g. peroxidized lipids) are generally toxic. It is therefore not surprising that plants have evolved large gene families for detoxification of ROS and their by-products. At least 148 enzymes have been defined as part of the ROS scavenging network of Arabidopsis [33]. However, of the 75 ROS network genes with sufficient hybridization signal intensity to be detected on our microarray, a majority of the responsive transcripts were down-regulated > 2-fold by NaCl (Table 2). The predominance of non-responsive or down-regulated transcripts was evident within almost every category of ROS-scavenging enzymes, except for alternative oxidase, ferritin, and monodehydroascorbate reductase, in which all NaCl-responsive transcripts were induced. The limited proportion of genes with NaCl-inducible transcripts within the ROS-scavenging network points to a considerable amount of sub-functionalization at the regulatory and catalytic levels, as well as roles for these genes beyond detoxification of ROS [34]. A third family of peroxidases, namely the type III (heme-containing) peroxidases, is not usually considered part of the ROS scavenging network and may have diverse roles including generating ROS for cell wall modification [35]. We arbitrarily selected three peroxidase genes PER21 (At2g37130), PER69 (At5g64100), and PER27 (At3g01190), for validation by qRT-PCR, and confirmed that these were generally down-regulated by NaCl-treatment (Table 1). These observations are consistent with functions for type III peroxidases in biological processes that are largely distinct from ROS scavenging.

A large proportion of glutathione-S-transferases (GSTs) were induced by NaCl treatment (Table 2). We note that nearly all of the NaCl inducible GSTs are from the Tau family of GSTs, highlighting a distinct role for this sub-class of genes in oxidative stress responses, as has been previously inferred from proteomic studies [36].

### Transporters

The regulated transport of molecules across the plasma and vacuolar membranes is a well characterized response to abiotic stress [26]. Within the NaCl-responsive transcripts on our microarray, we observed enrichment of transporters for water, sugars, cations, and other molecules (Table 2). The NaCl-responsive aquaporins and vacuolar-type ATPases detected on our microarray were enriched almost exclusively in down-regulated transcripts. In contrast, almost all of the detectable NaCl-responsive Na^+^/H^+ ^exchangers were induced by stress. The homogeneity of these responses indicates relatively little sub-functionalization within these groups of genes, as compared to the ROS scavenging network described above. Among the remaining antiporters, as well as sugar transporters, ABC (ATP-binding cassette), and LeOPT1-like transporters and MATE-like (multi-antimicrobial extrusion) efflux carriers, specific transcripts were induced and others were repressed by NaCl-treatment (Table 2) [37,38]. Within the MATE family in particular, the proportion of NaCl-inducible transcripts is relatively large, as transcripts representing approximately half of the 26 detectable genes were induced > 2 fold by NaCl, while only one MATE gene was equivalently repressed. One of these transcripts was among those we selected for further confirmation by qRT-PCR (Table 1). Both qRT-PCR and microarray analysis showed an approximately 8-fold increase in transcript abundance after 6 h of NaCl treatment, with further accumulation to a 32-fold increase by 48 h. The large proportion of induced genes within the MATE family, plus the magnitude of their induction, suggests a previously underappreciated role for MATE-efflux carriers in the root response to NaCl. Very little is known about this family of carriers, although a previous meta-analysis of microarray data identified some MATE transporters among a list of stress-induced transporters [39].

### Primary energy metabolism, carbohydrates and cell walls

Transcripts required for the main respiratory pathways of glycolysis, the tricarboxylic (TCA) cycle, and the pentose phosphate pathway (PPP) were generally non-responsive or were down-regulated by NaCl treatment (Table 3). Genes encoding components of the mitochondrial electron transport chain were especially enriched among down-regulated profiles (Table 3). The down-regulation of these energy-evolving pathways is a common stress response that may serve to conserve energy and limit growth and further generation of ROS [40]. The few glycolytic and TCA-related transcripts that were up-regulated were all isoforms of other genes that had been down-regulated, although no correlation between cytoplasmic location and NaCl-response could be detected (data not shown). Transcript abundance for almost all components of the PPP was also decreased (Table 3), including D-ribulose-5-phosphate 3-epimerase (At3g01850, At1g63290), ribose 5-phosphate isomerase (At2g01290, At3g04790), 6-phosphogluconolactonase (At5g24410, At3g02360), and glucose-6-phosphate-1-dehydrogenase (G6PDH) (Additional File 1). The PPP is an important source of NADPH, especially in non-photosynthetic tissues, and therefore might be expected to have increased activity under oxidative stress [41-43]. However, we noted that the down-regulated G6PDH transcripts we detected were G6PD5 (At3g27300) and G6PD6 (At5g40760), which are the major, cytosolic versions of G6PDH expressed in Arabidopsis roots and are distinct from the four genes encoding plastid-localized isozymes [44]. Previous analyses of G6PDH enzyme activity in potato and tobacco leaves has shown that isozymes located in plastids, but not the cytosol, have increased enzymatic activity following exposure to osmotic and oxidative stress [45,46]. Thus, G6PD5 and G6PD6 may function preferentially under conditions other than osmotic and oxidative stress. The sub-functionalization of isozymes into groups with contrasting responses to stress appears to be a common theme within the PPP, TCA, and glycolysis-related genes detected on our array.

Glycosyltransferases (GTs) and glycoside hydrolases (GHs) are two large superfamilies of carbohydrate-active enzymes [47]. All GTs catalyze similar biochemical reactions, namely the transfer of sugar moieties to acceptor molecules. Conversely, GHs break bonds existing between sugar moieties other molecules. Within each of these superfamilies, a significant and roughly equivalent number of genes were either up-regulated or down-regulated by NaCl at the transcript level, suggesting dynamic regulation of glycosylation in response to stress. These salinity responsive genes were distributed throughout the many constituent gene families of GTs and GHs, such that only two of the constituent families of GHs were significantly enriched in NaCl-responsive genes by our criteria: GH16, and GH28 (Table 3.). One member of the GH16 family, *XYLOGLUCAN ENDOTRANSGLUCOSYLASE/HYDROLASE 18 *(XTH18; At4g30280), was previously reported to be expressed in the base of elongating roots, and is among the NaCl-induced genes we selected for validation by qRT-PCR (Table 1) [48]. GH16 and GH28 are comprised largely of xyloglucanases and galacturonases, respectively, which are both often associated with cell wall metabolism. A limited number of other cell-wall related gene families were also enriched in NaCl-responsive transcripts, most notably the expansins (Table 3) [49], for which at least one-third of the genes were transcriptionally up-regulated by NaCl. Increased activity of expansins and xyloglucanases is consistent with the increase in cell wall flexibility observed in response to osmotic stress in some species [50].

A decrease in transcript abundance for many enzymes involved in lignification, as well as the potentially cell wall-related lipid transfer proteins (LTPs), were also observed in our microarray data (Table 3). However, we note that two families of enzymes that are sometimes grouped in the "lignification tool box", namely the 4CL (4-coumarate:CoA ligase)-like and the COMT (Caffeic acid O-methyltransferase)-like families, were almost entirely up-regulated in response to NaCl stress [51]. It is should be emphasized that these two families do not include their namesakes *4CL *and *COMT*, which are enzymes with clearly established roles in lignification. Thus the 4CL-like and COMT-like genes may have unique roles in the NaCl stress-response, and may not necessarily be related to lignification. LTPs are another group of proteins, some of which have been shown to affect cell wall extensibility or to be secreted in response to NaCl stress [52,53]. Nearly half of the detectable LTP transcripts on our microarray were down-regulated by NaCl treatment (Table 3), although at least 5 LTPs were up-regulated. It is likely that these various LTPs represent a diversity of stress responses, some of which may also be related to cell-wall extensibility.

### Protein metabolism

The impact of abiotic stress on protein metabolism is evident in our microarray results. A decrease in bulk protein translation following NaCl has been detected in proteomic studies, and is consistent with our observed down-regulation of the majority of transcripts for almost all cytosolic and plastidic ribosomal proteins (Table 4) [54]. Among proteins that promote the proper folding of proteins and/or prevent the aggregation of nascent or damaged proteins, we detected enrichment of NaCl-responsive genes within several gene families. Within the peptidyl prolyl isomerase super-family, the 29 genes represented by detectable transcripts were largely down-regulated by NaCl (Table 4) [55], although only the cyclophilins were significantly (< 5% FDR) enriched in down-regulated transcripts. A second superfamily of genes related to protein folding and aggregation are the heat shock proteins (HSPs) [56,57]. As expected, a majority of the 51 detectable HSP superfamily genes were transcriptionally induced by NaCl, and transcripts for only 5 genes were down-regulated by > 2 fold (Table 4). Although the majority of HSPs are resident in the cytosol, most of the 5 down-regulated genes detected in the HSP superfamily were targeted to specific compartments, including two genes targeted to the endoplasmic-reticulum (ER) (HSP70, At4g16660; HSP90-like *SHEPERD*, At4g24190), a chloroplast targeted HSP100/CLP (At5g50780). Consistent with these observations, some HSP family proteins that are targeted to chloroplasts or the ER have been previously shown to be repressed by particular abiotic stresses [58].

Proteolytic activity has been attributed to over 600 peptidases within the plant genome, [59]. In our microarray data, both up- and down-regulated transcripts were detected for genes within each of the aspartic, cysteine, serine, and metallo-peptidase super-families (Table 4). Specific families and sub-families within the cysteine and serine peptidases were significantly enriched in NaCl responsive genes, as shown in Table 4. In contrast to the other peptidase superfamilies, the responsive threonine peptidases that comprise the 20S catalytic core of the 26S proteasome were exclusively down-regulated. A predominance of down-regulation was also observed among the transcripts comprising the other component of the 26S proteasome, namely the 19S regulatory particle (Table 4). However, many of the gene families putatively encoding functions upstream of the 26S proteasome in the ubiquitination pathway were up-regulated. Notably, SKP1 (S-phase-associated kinase protein) kinases, E3 U-box proteins, and E3 RING (Really Interesting New Gene) finger proteins were all significantly enriched in NaCl-induced genes (Table 4). Because the 26S proteasome works in conjunction with the ubiquitination pathway as the major proteolytic system of the nucleus and cytosol, it appears paradoxical that many ubiquitination-related proteins would be up-regulated, while the detectable 26S proteasome transcripts would be uniformly down-regulated by NaCl treatment [60]. This suggests a role for some SKP1, E3 U-box proteins, and E3 RING related enzymes that is independent of the 26S proteasome in the stress response, and may also hint at aspects of regulatory complexity not captured by microarray analysis [61].

### Signal transduction components and hormones

The predicted kinases of Arabidopsis form a very large superfamily of at least 979 genes that has been subdivided into 5 classes comprised in total of 81 families [62,63]. Nearly all of the NaCl-responsive kinases on our microarray were associated with the two largest classes in the PlantsP classification system (PPC:1, PPC:4) and twelve families therein (Table 5). These include eight families of receptor-like kinases (RLKs) and related kinases: legume lectin domain (LLD), leucine rich receptor (LRR) II & X, and LRR XI &XII, receptor like cytoplasmic kinase (RLCK) VII, wall-associated kinase (WAK)-like, proline-rich extensin-like kinases (PERK), S-Domain (type 1), and domain of unknown function (DUF) 26. Within each of these families, nearly all NaCl-responsive genes increased in transcript abundance following stress-treatment, and none of the detectable PERKs or S-Domain (type 1) kinases was repressed by NaCl (Table 5). Aside from the RLKs and related proteins in class PPC:1, we also detected enrichment of NaCl-responsive transcripts within several families of non-transmembrane kinases, including Mitogen Activated Protein Kinase Kinase Kinases (MAP3Ks), Calcium Responsive Kinases (CRKs), Ribosomal Protein S6 Kinases (RPS6K), and an unnamed family (PPC:4.4.1) of kinases. Among these, up-regulated transcripts were especially predominant in the RPS6K and PPC:4.4.1 families.

Protein phosphatases are divided into three major classes: Protein Serine/Threonine Phosphatases (STKs), Dual Specific Phosphatases (DSPs), and Protein Phosphatase 2C (PP2C) [62]. We observed that the STKs and DSPs differed greatly from the PP2Cs in their transcriptomic response to NaCl-shock (Table 5). Transcripts for only one of the 18 detected STKs or DSPs were strongly induced by NaCl treatment, whereas transcripts for more than half of the detectable PP2Cs were strongly induced by NaCl. Some PP2Cs have been previously identified as components of the ABA signalling pathway, and all of these are among the NaCl-induced PP2Cs detected on our array: *ABI1 *(*ABA INSENSITIVE 1*; At4g26080), *ABI2 *(At5g57050), *HAB1 *(*HOMOLOGY TO ABI1/ABI2*; (At1g72770), *HAB2 *(At1g17550), and *AHG3 *(*ABA-HYPERSENSITIVE GERMINATION3*, At3g11410) [64-67]. Our data indicate that nearly twenty other PP2Cs, including those outside of the ABI/HAB clade defined by Saez and colleagues, may have important roles in NaCl stress signalling.

The Two-component signal transduction system, which consists of histidine protein kinases (HKs), His-containing phosphotransfer proteins (HPs) and response regulators (RRs), is involved in plant hormone, stress, and light signaling [68]. The Arabidopsis genome contains 54 genes encoding putative HKs, HPs, RRs, and related proteins. In our data, none of the 16 HKs were induced by salt, while *AHK1*, a putative osmosensor [69], was down-regulated at the 6 h time-point (see Additional File 1). However, the A-type RRs were significantly enriched among clusters of NaCl-induced genes (Table 5). These include *ARR5*, *ARR6*, and *ARR7*, which act as negative regulators to repress the expression of the cytokinin-responsive genes [70], suggesting a potential link between NaCl stress and cytokinin signalling.

A handful of other families of putative regulatory proteins were statistically enriched among clusters of NaCl-responsive genes in our analysis. Transcripts for at least half of the six MLO (mildew resistance locus o) proteins detected on our microarray were induced by NaCl-treatment [71]. These are seven-transmembrane domain proteins with features similar to G-protein coupled receptors, and some members of this gene family have been reported to be abiotic stress responsive [72]. Finally, transcripts decreased for at least 5 of the 8 14-3-3 genes detectable in our root microarray, while no significant increase in transcript abundance for any 14-3-3 protein genes was observed [73].

Ethylene and jasmonic acid (JA) are hormones whose activity has been previously correlated with environmental stress [16,74,75]. The rate-limiting step of ethylene biosynthesis is the production of 1-aminocyclopropane-1-carboxylic acid (ACC) by ACC synthase (ACS), which is followed by the conversion of ACC to ethylene by ACC oxidase (ACO) [76]. ACS and ACO are each encoded by gene families. Four ACC synthase and one ACC oxidase genes were significantly up-regulated by NaCl-treatment, while another 3 ACO genes were down-regulated by salt. Differential regulation of members of the ACO gene family indicates that although ACS is the key regulatory point for ethylene biosynthesis, regulation of ACO expression also has functional significance [76]. Ethylene signalling transduction involves ethylene response factor (ERF)/ethylene response element binding protein (EREBP), which have been shown to act as activators or repressors of additional downstream ethylene-responsive genes. JA and its derivatives are also known to be involved in a wide range of developmental processes, as well as stress and defence responses [75]. At least 7 genes implicated in the biosynthesis of JA were induced by salt stress (Table 5), including *LIPOXYGENASE3 *(*LOX3*, At1g17420), *ALLENE OXIDE CYCLASE *(*AOC1*, At3g25760), *AOC2 *(At3g25780), and *ALLENE OXIDE SYNTHASE *(AOS, At5g42650), indicating that biosynthesis of this hormone is also NaCl responsive.

Within the biosynthetic pathway for indole acetic acid (IAA), which is an auxin, we detected up-regulation of two genes in response to NaCl treatment: namely nitrilases *NIT1 *(At3g44310) and *NIT2 *(At3g44300) (Table 5) [77]. Consistent with an increase in levels of bioactive auxin, we also detected down-regulation of transcripts for *SUPERROOT2 *(*SUR2*, At4g31500), an auxin conjugating enzyme [78]. We confirmed the repression of *SUR2 *transcripts by qRT-PCR (Table 1). Furthermore, we noted that of 4 putative auxin efflux carriers detectable after hybridization, transcripts for *PIN1 *(At1g73590), *PIN3 *(At1g70940), and *PIN7 *(At1g23080) were induced by our NaCl treatment [37]. Part of the increased auxin activity we infered from our microarray data may be directed towards increased lateral root formation, which is an auxin-regulated, and PIN requiring process that has been previously described in NaCl-treated roots [16,79]. Lateral root formation also involves genes from the *LATERAL ORGAN BOUNDARIES *(*LOB*) family; consistent with this requirement, we observed that the class I sub-group of this family was exclusively enriched in up-regulated transcripts, with at least seven genes induced following NaCl-treatment (Table 5)[80]. Thus, the initiation of lateral root primordia is a NaCl-stress response that appears to be distinctly represented within our microarray data, even though the process involves only a small number of cells within the entire root.

### Transcription factors

The Arabidopsis genome has at least 1,819 predicted transcription factors (TFs), which have been classified into 56 families [81-83]. From the over 1,613 transcription factors represented as probes on our microarray, transcripts for 734 genes could be detected at a signal intensity above background. Of these, 289 predicted TFs were up-regulated and 139 transcription factors were down-regulated at least 2 fold at one or more time points following NaCl treatment (Table 6). STEM analysis identified 17 families that were significantly enriched among clusters of NaCl responsive genes. These TF families range in size from 203 to 7 members, and are described below. Although these 17 families contain almost two-thirds of the predicted TFs in the Arabidopsis genome, it is worth noting that transcripts for several other relatively large TF families were detected on the array, but these were largely unresponsive to NaCl stress. Some of the notable TF families that were expressed in roots, but which were not significantly enriched in NaCl-responsive transcripts are: MADS (MCM-1, Agamous, Deficiens and Serum Response Factor), ARF (Auxin Response Factor), Nin-like (nodule inception), and TUB (Tubby).

At least four TF families have been reported to be enriched in stress-responsive genes: MYB (Myeloblastosis), HSF (Heat Shock Factor), AP2/EREBP (Apetala-2/EREBP), and WRKY (named after the WRKY amino acid motif). A recent analysis of MYB-transcription factor gene expression concluded that almost all MYB TFs are responsive to stresses or hormones [84]. Consistent with this report, we observed that at least one third of the 84 detectable MYB TFs were induced by NaCl at the transcript abundance level, and only a small fraction were repressed (Table 6). MYB15 (At3g23250), which is a R2R3-type MYB gene, was induced approximately 16-fold by NaCl at each of the three time points [85], and we confirmed this by qRT-PCR (Table 1). Similarly, most of the HSF TFs detected on our array were up-regulated by NaCl, as expected for this family of stress-inducible TFs [86](Table 6). The AP2/EREBP family includes some of the best characterized stress-responsive transcription factors, including the CBF (CRT/DRE binding factors) proteins [87,88]. We observed NaCl-inducible transcript accumulation in more than half of the 76 AP2/EREBP TFs detectable on our array (Table 6); and these up-regulated transcripts were detected in each of the AP2/EREBP family's subgroups (i.e. AP2, RAV, ERF I-X) [89]. We arbitrarily selected one AP2/EREBP TF (At1g44830, TINY-related) for confirmation by qRT-PCR. As shown in Table 1, both microarray and qRT-PCR analyses were in agreement about the strong inducibility of transcripts of At1g44830 following NaCl-treatment. The majority of WRKY family TF genes are known to be responsive to biotic and/or abiotic stress, although most research has focused on the role of these genes in pathogen responses [8,90-92]. We observed that transcripts for 18 of the 35 WRKY TFs detected increased by at least 1.5 fold in response to NaCl treatment. We used qRT-PCR to confirm these observations for *WRKY17 *(At2g24570), *WRKY25 *(At2g30250) and *WRKY33 *(At2g38470), which are three of the least studied members of this family. Both microarray and qRT-PCR confirmed that *WRKY17 *and *WRKY33 *transcripts increased at least 14-fold following NaCl treatment, although *WRKY17 *transcript abundance peaked at 6 h and gradually diminished, whereas *WRKY33 *abundance remained very high throughout the time points sampled. The transcript level of *WRKY25 *also increased and peaked at 48 h with an induction of at least 22 fold. These observations emphasize a potentially important role for WRKY transcription factors in abiotic stress responses.

Many TFs contain zinc-finger motifs including all members of the following families: C2H2 (Cys2- His2), C2C2 (Cys2- His2), C3H (Cys3- His) LIM (Lin-11, Isl-1 and Mec-3), PHD (Plant Homeodomain), WRKY, ZF-HD (zinc finger homeodomain), and ZIM (Zinc-finger protein expressed in Inflorescence Meristem). Of these, we observed the WRKY (as described above), and C2C2, C2H2, and ZIM families were particularly enriched in NaCl-responsive transcripts. The C2C2 and C2H2 families are relatively large, and had significant numbers both of up-regulated and down-regulated genes. The smaller ZIM family was represented by 10 genes with detectable transcripts in our analysis; 8 of these genes were up-regulated by NaCl treatment. Knowledge about the biological role of ZIM TFs is very limited. Previous studies have shown a putative relationship of some ZIM family members with GA-independent cell elongation in shoot tissues [93,94]. The large proportion of ZIM family members with NaCl-inducible transcripts makes this group of TFs another attractive target for further functional characterization. We assayed the expression pattern of one ZIM transcription factor (At3g17860) by qRT-PCR and confirmed that it is induced after 6 h of NaCl, although the transcript abundance falls off rapidly after 24 h and 48 h (Table 1).

Four other large families of TFs, each with distinct DNA binding motifs, are: bHLH (basic helix-loop-helix), bZIP (basic leucine zipper), NAC (NAM, ATAF1,2, CUC2), and HB (homeobox) genes. All of these families contained NaCl-induced genes, although only the HB and NAC families were significantly enriched in NaCl-responsive transcripts. We selected one bHLH TF (*bHLH092*, At5g43650) and one NAC TF (At4g01550) for further confirmation by qRT-PCR. The NAC domain TF was chosen because its expression peaked at later time points than most TFs; *bHLH092 *was selected because it was among the mostly highly induced transcripts of any gene on our microarray. Furthermore, this bHLH is not represented among probes on Affymetrix arrays, and therefore would not be detected in some previous transcriptomic studies, although it was among the NaCl-inducible transcripts identified in a long-oligomer microarray analysis of whole Arabidopsis plants [9]. Our qRT-PCR analysis showed a NaCl-dependent increase of up to 50-fold for the NAC TF (At4g01550) transcripts, and an increase for *bHLH092 *of over several magnitudes (Table 1). Clearly, these two TFs, and many of the other NaCl-responsive TFs shown in Table 6 are potentially important regulators of the NaCl-stress response in Arabidopsis roots.

An interesting property of the groups of transcription factors described in Table 6 is that the majority of the families (34/39) contain members that are assigned to different expression profiles. In many cases, related transcription factors show very divergent expression patterns, with some members of a single gene family induced early in the stress response, while other members are induced only at subsequent time points. For example, while the majority of AP2/ERF family members have a peak relative expression ratio at 6 h (Table 6, and Figure 2, STEM profile b), some AP2/ERF transcripts had peak expression ratios at 24 h (profile f), or increased in abundance throughout the experiment until at least 48 h (profile a), while other genes did not show a significant relative change in expression until 48 h (profile h), or showed a more complex pattern of regulation (profiles c, d, e), or were predominantly down-regulated (profiles i, j, k, l, o). Similarly divergent patterns of transcriptional responses have been described previously within families of oxidative stress inducible genes [95]. The large number of up- or down-regulated transcription factor genes we detected, as well as the diversity of their temporal expression patterns, is consistent with the existence of a highly complex regulatory network underlying the physiological response to NaCl treatment.

## Conclusion

Our observations of the transcriptomic response to NaCl-treatment in Arabidopsis roots were consistent with the broad themes of abiotic stress responses previously reported in other systems [26]. However, our functional enrichment analysis showed that a considerable amount of sub-functionalization has occurred within many gene families, demonstrating the complex relationship between stress and processes related to reactive oxygen species, cell wall growth, transport, signal transduction, hormone signalling, proteolysis and transcriptional regulation. Moreover, STEM enrichment analysis allowed us to detect previously underappreciated families within these broad categories that are strongly associated with stress response, including MATE transporters, LeOPT1-like transporters. PERK kinases, MLO-like receptors, carbohydrate active enzymes (e.g. XTH18), transcription factors, and other proteins (e.g. 4CL-like, COMT-like, LOB-Class 1), and verified the expression of a selection of these genes by qRT-PCR. These data will facilitate the mapping of root regulatory networks and better understanding of stress phenomena in plants.

## Methods

### Plant growth and stress treatment

We sowed surface-sterilized seeds of *Arabidopsis thaliana *(ecotype Col-0) on (3.5 mm diameter) agar plugs arrayed on custom-made polypropylene rafts, and cold stratified them at 4°C for 2d. Each plug consisted of 1/2 × Murashige and Skoog (MS, Sigma) basal salts, 1% sucrose, and 0.7% phytagar, and each raft held approximately 30 seeds, spaced ca. 15 mm apart. Rafts were transferred into opaque plastic tanks containing 1 L of autoclaved 1/4 × MS solution (pH5.7) in a growth chamber at 22°C, 70% relative humidity, and light (125 μMm^-2 ^s^-1^) on a 16 h light/8 h dark cycle. The solution in the tanks was aerated and replaced with fresh, autoclaved 1/4 × MS solution every 4 days to limit growth of microorganisms. At 18 days post-germination (dpg), seedlings were treated with 1/2 × MS medium (pH5.7) supplemented with 150 mM NaCl beginning at 8:30 AM (1.5 h after the start of the light cycle), and roots were harvested at 6 h, 24 h and 48 h post-treatment, and were frozen in liquid nitrogen and held at -80°C. Untreated controls were harvested in parallel, at the same time points. Approximately 150 plants were harvested and pooled for each combination of time point and treatment, in each biological replicate. This process was repeated in three biologically and temporally independent replicates.

### Physiological analyses

Proline concentrations were measured using the method of Bates [96], with L-proline (Sigma) as a standard. Anthocyanin concentrations were determined according to Macinelli and Schwartz [97]. For the root elongation assays, 5 dpg seedlings grown on solid growth medium (1/2 × MS, 2% sucrose, 0.7 % phytagar) were transferred to square petri dishes containing similar growth medium supplemented with 150 mM NaCl. Root elongation was measured 5 days after transfer.

### Microarray preparation, hybridization and data extraction

We deposited 26,090 70 mer oligonucleotide probes (Array-Ready Oligo Set for Arabidopsis genome Version 1.0 was from Qiagen Operon), plus additional probes for quality control on superamine aminosilane-coated slides (Telechem). Probe spotting followed previously described methods [98,99]

Total RNA was isolated from both control and stressed root tissue by RNeasy Midi kit (Qiagen), and was concentrated by Microcon YM-30 (Millipore) to be ~1.2 μg/μL and quantified by NanoDrop ND-1000 Spectrophotometer. RNA integrity was checked on a formamide denaturing agarose gel. 5 μg total RNA was used to synthesize cDNAs using SuperscriptII (Invitrogen) with RT polyA-capture primers in 3D Array900 kit (Genisphere). A two-step hybridization was performed, i.e., cDNA from both control and treated tissue were hybridized together at 55°C overnight under a 24 × 60 mm cover slip (Fisher Scientific) in a sealed humid 50 ml centrifuge tube. Secondary hybridization with Cy3 and Cy5 dendrimers were performed at 55°C for 4 h and washes were done according to the manufacture's protocol. Slides were scanned immediately using ArrayWorxe (Applied Precision) and transformed into tiff images. To avoid bias in the microarray evaluation as a consequence of dye-related differences in labelling efficiency and/or differences in recording fluorescence signals, dye labelling for each paired sample (stressed/control) was reversed in two individual hybridizations. Therefore, two hybridizations were performed for each of three independent biological replicates at each time point.

### Microarray data analysis

Background-subtracted spot intensities were measured by Spotfinder v3.0, and normalized by the Loess method in MIDAS v2.19 [100]. Spots were defined as detectable above background if their signal intensity in at least on channel was greater than two standard-deviations above the mean signal intensities of all blank and randomized negative control spots, and if they were detectable in at least two-thirds of arrays at any one time point. Fold changes (treated/control) were calculated in log2 scale, and significance analysis of microarrays (SAM) was applied to find genes for which the fold change differed significantly from zero in a multiclass test (with inferred values at time point 0), using a false discovery rate (FDR) of ≤5% [21]. Assignment of genes to temporal expression profiles and detection of statistically enriched gene families within each profile was conducted using STEM (Short Time Series Expression Miner) software, with maximum number of model profiles set to 16, and maximum unit change set to 3 [22]. The input data for STEM analysis consisted of all normalized, intensity-filtered data points identified by SAM analysis as statistically distinct from 0. The p-values derived from STEM analysis were corrected for multiple hypothesis testing using a FDR of < 5% [101]. The raw microarray data of 18 hybridizations as well the protocols used to produce the data were deposited in the ArrayExpress database under the accession number E-MEXP-754.

### Real-Time quantitative PCR

Aliquots of the same RNA samples used for microarray analysis were also used for real-time PCR. Reverse transcription (RT) was performed with 2 μg of total RNA pretreated with DNase I (Ambion) to obtain cDNA with SuperScript II and Oligo(dT)_12–18 _(Invitrogen) as the primer in a 20 μL reaction volume. Each cDNA sample was diluted 1:4 in sterile ddH_2_O, and 1 μL of this dilution was used as template for qPCR. Primers for the PCR reactions were designed by PrimerSelect 5.00 of DNAStar (DNASTAR Inc) to have a Tm of ~ 60°C and an optimal annealing temperature of 53–55°C with the length of the amplicons between 80 and 200 bp. Real-time PCR was performed with QuantiTech PCR SYBR Green I Kit (Qiagen) in 20 μL reactions using the LightCycler 1.5 System (Roche Diagnostics) according to the manufacturer's instruction. Each PCR reaction contains 1 μL of cDNA, 0.5 μM of each of the primers and 10 μL of master mix. The initial denaturing time was 15 min at 95°C, followed by 35 cycles consisting of 94°C for 15 s, 53°C for 30 s, and 72°C for 20 s with a single fluorescence measurement. A melting curve (65°C–95°C with a heating rate of 0.05°C s^-1 ^and a continuous fluorescence measurement) was run after the PCR cycles followed by a cooling step at 40°C.

For relative quantification, amplification efficiencies (E) for each gene were determined as follows: a portion of cDNAs transcribed from 5 μg of total root RNA was diluted with sterile ddH_2_O to be 10^-1^, 10^-2^, and 10^-3^. Standard curves for each gene was performed using the original and diluted cDNAs to cover the range of all template concentrations. Real-time PCR efficiencies (*E*) were calculated from the given slopes in the LightCycler software of the standard curves according to the equation: *E *= 10 ^-1/slope^. Crossing points, defined as the point at which the fluorescence rises above the background fluorescence was determined using the "Fit Point Method" in the LightCycler software 3.5.3 (Roche Diagnostics). Gene-specific PCR efficiency was used to calculate the expression of target genes relative to the expression of UBQ10 reference gene.

The genes tested for expression levels by quantitative PCR were as follows:

At1g44830 (AP2/EREBP), forward primer (FP), 5'-AAGTTGGGGTTCATGGGTTTCA-3', reverse primer (RP), 5'-ACATAGGAGTGCTGCGTCGTAGG-3';

WRKY17 (At2g24570), FP, 5'-CAGCGAGGAAACACGTGGAAAGAGC-3', RP, 5'-CAAGCCGAACCAAACACCAAACCAC-3';

MYB15 (At3g23250), FP, 5'-AAATACTTGGCAATAGATGGTCAG-3', RP, 5'-ACGGATTCAGATTTTGGTTTAGTA-3';

XTH18 (At4g30280), FP, 5'-AGGCTCCTTTCACCGCTTTCTA-3', RP, 5'-ACTCTGTACCCCTTTCATTCTTGTCTG-3';

At4g01550 (NAC), FP, 5'-AGAGAAGAAGAGCTGGTTTATTACA-3', RP, 5'-TTCATCGCCGGGTTCAAGT-3';

At2g04070 (MATE), FP, 5'-TCTTTCTGGTTTTTCGCTATGACACT-3', RP, 5'-ATTGCCGCTGATGGGACTC-3';

At4g26410, FP, 5'-GAGCTGAAGTGGCTTCCATGAC-3', RP, 5'-'GGTCCGACATACCCATGATCC-3';

CYP83B1/SUR2 (At4g31500), FP, 5'-ACTCTTGACCC TAACCGCCCTAAAC-3', RP, 5'-TGCAGCCGCCGTGTCAGT-3'.

bHLH92 (At5g43650), FP, 5'-CACGGAACTGAGGATGATGTC-3', RP, 5'-GTCGCCGGCTTCTTTCCTTCTCT-3'.

MYB15 (At3g23250), FP, 5'-AAATACTTGGCAATAGATGGTCAG-3', RP, 5'-ACGGATTCAGATTTTGGTTTAGTA-3';

WRKY25 (At2g30250), FP, 5'-TGGTTCTTCCGGCGTTGACTGTTA-3', RP, 5'-GTGAAATCGGAAGAGGTGGTGGTTG-3';

At3g17860 (ZIM), FP, 5'-GGCTCCAACAGTGGCATTACCTCT-3', RP, 5'-TATGGGGATACGCTCGTGACCCTT-3';

PER21 (At2g37130), FP, 5'-TGCGAGAGACGGTATTGTCA-3', RP, 5'-TCTCCCAAGTAGCTCCCTCT-3';

PER27 (At3g01190), FP, 5'-CGCAATGGTTGCACTTGA-3', RP, 5'-TGAGCGAAAATCGCTGATAA-3';

PER69 (At5g64100), FP, 5'-CTCTTGTTGGCGGACACA-3', RP, 5'-GTCGATTGATGGGTCAGGTT-3'.

The internal control gene was UBQ10 (At4g05320), FP, 5'-TAC TCT TCA CTT GGT CCT GCG TCT TC-3', RP, 5'-GGC CCC AAA ACA CAA ACC ACC AA-3'.

qPCR for each gene was done on 3 biological replicates with duplicates for each biological replicate. The relative transcript level was determined for each sample, normalized using the UBQ10 cDNA level, and averaged over the 6 replicates.

## Authors' contributions

YQJ carried out all experiments and drafted the manuscript. MKD participated in the design of the study and assisted with analysis and manuscript preparation.

## Supplementary Material

Additional File 1 **Microarray expression ratios for all probes significantly responsive to NaCl, and comparisons to previously published data. **Probes are shown which, after one or more of the three time points following 150 mM NaCl treatment, (i.e. 6 h, 24 h, or 48 h), had a fold change in expression ratio (treated:control) that different significantly from 0 (log_2_), according to SAM analysis (FDR < 5%) [21]. All probes were also filtered to remove those with low signal in both channels of more than 1/3 of the arrays at each time point, as described in Methods. Blank cells indicate that data has been removed due to filtering. Only probes with associated AGI identifiers are shown, and some AGI loci are represented by more than one probe. The expression ratios (log_2_, treated:control) for each of 18 hybridizations (3 biological replicates by 2 dye-reversal replicates at each of the 3 time points) are shown in columns 5–22, and can be distinguished by the column headers. The mean expression ratio at each time point is given in log_2 _scale in columns 23–25. Rows are sorted in decreasing order, according to the mean of all expression ratios in each row as shown in column 26. Microarray expression data was extracted from selected previous publications, and is summarized in columns 27–39, wherever genes common AGI numbers could be identified. All quantitative data is expressed as (log_2_, treated:control) expression ratios. Columns 27–28 show expression ratios for all transcripts present on Affymetrix ATH1 arrays hybridized to 150 mM NaCl treated roots (6 h, 24 h), as described in the unpublished AtGenExpress data set prepared by the Kudla group [15]. Columns 29–30 show expression ratios only for up-regulated genes identified in NaCl treated roots (3 h, 27 h) measured on the 8,100 gene Affymetrix GeneChip, as described in Supplemental Table 1 of data prepared by Kreps and colleagues [12]. Column 31 shows expression ratios for genes identified as being up-regulated in roots after 2 h of 250 mM NaCl treatment, as reported in Table 2 of a report by Taji *et al*. [10]. These, as well as other relevant reports that lacked quantitative data are summarized qualitatively in Columns 32–39. Wherever our present, spotted microarray data and the Kudla AtGenExpress data showed similar patterns of expression for a given gene (i.e. up- or down-regulated by 2 or more fold in both data sets at a specific time point), this is indicated by the symbol 'Y' in the appropriate row of columns 32 or 33. Columns 34–39 contain the symbol 'Y' if the gene in that row was included in the lists of up-regulated genes reported in the following sources: Kreps et al. [12] (roots, 150 mM; 3 h, Column 34; 27 h, Column 35), Taji et al. [10] (roots, 250 mM, 2 h, Column 36), Maathuis et al. [13] (transporters only, unspecified tissues and NaCl concentration, Column 37), Kamei et al. [9] (Tables 1, 3, 5 of original report, genes up-regulated after 2 h or 5 h 250 mM treatment of *sos*2 or *sos*3 mutants, Column 38), Ma et al. [5] (Table 2 of original report, root-specific NaCl induced gene expression, Column 39). Click here for file

Additional File 2 **Assignment of genes to functional categories described in text. **AGI identifiers and corresponding categories for the pathways or gene families named in Tables 2, 3, 4, 5, 6 of the manuscript. Authorities for assignment of AGIs to each category are cited in the respective legends of Tables 2, 3, 4, 5, 6. All genes in each category are listed here, regardless of their status in our microarray data. Some categories are further divided into sub-categories, which are denoted by double colons (::). This two column file may be used directly as input in STEM software [22]. Click here for file

## Figures and Tables

**Table 1:** Comparison of qRT-PCR and microarray results for selected genes.

AGI#	annotation	microarray (log2 ratio)			qRT-PCR (log_2 _ratio)		
		6 h	24 h	48 h	6 h	24 h	48 h

At1g44830	AP2/ERF	4.37	5.42	4.89	6.05	6.37	6.47
At5g43650	bHLH	7.21	7.58	7.45	11.05	11.93	11.87
At4g31500	CYP83B1/SUR2	-2	-1.89	-1.43	-1.84	-1.6	-1.22
At4g26410	expressed protein	0.01	0	0.04	-0.3	-0.23	-0.27
At2g04070	MATE	3.77	5.47	5.35	3.1	4.92	5.27
At3g23250	MYB15	4.05	3.25	1.31	5.39	5.67	5
At4g01550	NAC	1.93	3.35	3.21	2.46	4.87	5.65
At2g37130	PER21	-1.73	-1.56	-0.98	-1.32	-1.4	-0.69
At3g01190	PER27	-0.37	-0.02	0.14	-2.96	-1.21	-0.18
At5g64100	PER69	-1.78	-2.69	-1.8	-1.85	-2.4	-2.2
At2g24570	WRKY17	3.74	1.94	1.16	3.84	2.15	1.64
At2g30250	WRKY25	2.59	4.04	4.49	3.49	6	6.23
At2g38470	WRKY33	4.02	4.42	3.58	4.81	5.58	4.64
At4g30280	XTH18	2.54	1.3	1.57	2.65	1.58	2.5
At3g17860	ZIM	2.28	0.65	-0.84	2.42	1.02	-0.97

Genes representing a variety of expression patterns and functional categories were selected from the microarray dataset for further validation using qRT-PCR. Values shown are the mean of at least four independent measurements (microarray data), or three independent measurements (qRT-PCR).

**Table 2:** NaCl-responsive transcripts related to osmoprotection, detoxification, and transport.

	number of genes					
family, group, or pathway	in genome	on array	signal above bkgnd.	> 2 fold up	> 2 fold down	assignment to STEM profiles (Fig. 2) cluster:# of genes

osmoprotectants						
proline	5	5	5	2	1	a:1;b:1;j:1
trehalose biosynthesis*	22	21	15	8	1	a:4;b:4;c:1
trehalose phosphatase*	11	10	8	7	0	a:3;b:4
dehydrin*	10	10	6	4	1	b:1;c:2;h:1;i:1
						
ROS-response network						
alternative oxidase*	6	6	4	2	0	b:2
ascorbate peroxidase	9	8	6	1	1	b:1;h:1;o:1
blue copper protein	9	8	5	1	1	b:1;i:1;n:1
dehydroascorbate reductase	5	2	1	0	1	i:1
ferritin*	4	4	3	2	0	a:2
glutaredoxin*	27	26	8	3	3	a:3;i:1;j:2
glutathione peroxidase	8	8	4	0	1	j:2
glutathione reductase*	5	4	4	0	0	m:1
monodehydroascorbate reductase	5	5	4	2	0	a:1;c:1
NADPH oxidase*	10	10	4	1	2	c:1;i:1;k:1
NADPH oxidase-like	9	8	4	0	3	i:2;j:1
peroxiredoxin	11	10	5	0	2	j:2
superoxide dismutase	8	8	3	0	3	i:1;j:2
thioredoxins	32	31	20	5	7	a:1;b:2;c:1;f:1;i:2;j:3; l:1;o:1
						
class III peroxidase*	73	71	45	10	27	a:6;b:3;c:1;d:1;i:3;j:17; k:4;l:1;o:3
						
glutathione-S-transferase*	53	49	37	19	7	a:4;b:9;c:1;d:3;e:1;f:1; h:2;i:3;j:2;l:2;n:1;o:1
GST-tau family*	28	26	20	13	3	a:1;b:9;c:1;d:3;i:1;j:1; n:1
						
transporters						
aquaporin*	35	33	24	1	18	a:1;i:4;j:12;n:1
V-Type ATPase*	22	18	14	0	4	j:4
antiporter	70	66	25	5	9	a:4;c:1;i:2;j:4;k:1;l:2
Na+/H+ exchanger*	8	8	6	4	0	a:4
sugar transporter*	48	47	23	11	4	a:6;b:3;d:1;f:1;i:1;j:2; o:1
ABC transporter*	94	88	29	10	5	a:6;b:2;d:3;f:1;g:1;i:1; k:1;l:2
LeOPT1*	51	48	22	10	8	a:4;b:6;i:3;j:3;o:2
MATE*	56	51	26	13	1	a:4;b:6;c:1;d:1;f:1; i:1

Groups of genes that are significantly enriched in at least one STEM profile at a FDR of < 5% are marked with an asterisk (*). The final column details the frequency of assignment of individual genes to specific STEM profiles identified by corresponding letters in Figure 2. Functional categories in this table are defined according to the following sources: proline and trehalose biosynthesis [23]; dehydrins [27]; ROS-network [33]; class III peroxidases [25, 102], GSTs [25]; MATE transporters [38]; aquaporins [25, 103]; all other transporters [25, 37].

**Table 3:** NaCl-responsive transcripts related to respiration and carbohydrate and cell wall metabolism.

	number of genes					
family, group, or pathway	in genome	on array	signal above bkgnd.	> 2 fold up	> 2 fold down	assignment to STEM profiles (Fig. 2) cluster:# of genes

glycolysis*	70	64	45	6	10	a:2;b:1;d:1;f:1;h:2; i:3;j:5;k:3
tricarboxylic acid cycle	56	52	34	6	11	a:4;b:1;f:1;g:1; i:4;j:7
mitochondrial respiration*	95	66	55	0	26	g:1;i:2;j:23;o:1
non-oxidative pentose phosphate pathway	13	12	8	1	6	h:1;i:1;j:3;k:1;o:1
oxidative * pentose phosphate pathway	19	19	13	0	5	i:3;j:1;k:1
glycosyltransferase*	279	269	113	29	26	a:12;b:8;c:2;d:4;f:3; g:2;h:1;i:10;j:10;k:1; l:1;n:1;o:3
glycoside hydrolase (GH)*	306	289	121	42	27	a:17;b:16;c:1;d:4;e:3; h:2;i:11;j:6;k:2;l:2;o:3; p:1
GH family 16*	27	26	14	10	2	a:4;b:4;d:1;e:1;j:1; o:1
GH family 28*	52	46	10	4	1	a:3;b:1;d:1
expansins	35	32	21	7	4	a:1;b:3;c:1;d:2;i:3; m:1
lignification (exclusive of 4-CL like and COMT-like)	39	36	20	3	7	a:1;c:1;d:1;i:1;j:5; o:1
4CL-like*	9	8	3	2	1	b:1;d:1;k:1
COMT-like*	13	12	5	5	0	a:1;b:3
lipid transfer protein*	70	64	31	5	15	a:3;b:1;h:1;i:1;j:8; k:2;n:2;o:2

Groups of genes that are significantly enriched in at least one STEM profile at a FDR of < 5% are marked with an asterisk (*). The final column details the frequency of assignment of individual genes to specific STEM profiles identified by corresponding letters in Figure 2. Functional categories in this table are defined according to the following sources: glycolysis, TCA, electron transport and PPP [23]; all other groups: [25].

**Table 4:** NaCl-responsive transcripts related to protein metabolism.

	number of genes					
family, group, or pathway	in genome	on array	signal above bkgnd.	> 2 fold up	> 2 fold down	assignment to STEM profiles (Fig. 2) cluster:# of genes

ribosome large subunit*	121	100	83	0	59	i:19;j:36;n:4
ribosome small subunit*	94	87	74	0	53	i:24;j:24;n:1;o:1
ribosome plastidic	13	13	9	0	3	i:1;j:1;n:1
						
peptidylprolyl isomerase (PPI)						
PPI: cyclophilin*	28	28	19	1	9	b:1;d:1;i:1;j:8;l:1; m:1
PPI: FKB	23	22	7	1	2	a:1;j:2
PPI: parvulin	3	3	3	0	2	i:1;j:1
heat shock proteins*	70	68	51	36	5	a:31;b:3;c:1;f:1;i:2; j:2;o:1
peptidase*	557	524	265	43	73	a:20;b:12;c:1;d:4;f:6; g:4;h:1;i:16;j:46;k:3; l:2;n:2;o:6
aspartic peptidase	76	69	31	7	8	a:4;b:2;f:1;i:2;j:5;o:1
cysteine peptidase	102	102	55	11	15	a:5;b:2;d:3;f:3;i:4; j:7;k:2;l:1;n:1;o:1
family C26*	10	10	9	4	2	a:1;b:1;d:3;i:1;j:1
metallopeptidase	85	78	44	4	6	a:2;f:2;i:1;j:4
serine peptidase*	261	244	112	18	31	a:8;b:6;c:1;d:1;g:4; h:1;i:8;j:18;k:1;l:1; n:1;o:4
sub-family S10-004*	19	17	7	0	6	i:1;j:5
sub-family S10-005*	24	24	9	0	3	g:1;j:1;o:2
sub-family S33-UPW*	45	42	22	6	7	a:2;b:2;c:1;d:1;g:2; i:1;j:3;l:1;o:1
threonine peptidase *	28	28	22	3	13	a:1;b:2;i:1;j:12
20S proteasome subunit*	19	19	17	0	12	i:1;j:11
19S proteasome*	33	31	24	0	10	i:3;j:6
						
SKP1/ASK1*	17	16	9	5	3	a:2;b:1;h:2;j:3
E3 RING-type*	430	414	178	47	34	a:16;b:17;c:4;d:8;f:4; h:3;i:14;j:14;k:1;l:3;n:1; p:2
E3 Ubox-type*	61	53	30	16	3	a:6;b:8;c:1;d:1;j:2; o:1
Ubox class III*	12	11	9	7	2	b:5;c:1;d:1;j:1;o:1

Groups of genes that are significantly enriched in at least one STEM profile at a FDR of < 5% are marked with an asterisk (*). The final column details the frequency of assignment of individual genes to specific STEM profiles identified by corresponding letters in Figure 2. Functional categories in this table are defined according to the following sources: ribosomes [104] ; PPIs [55]; peptidases [59]; 19S proteasome [24]; E3 RING [105]; HSPs, SKP1s, E3 Ubox [25].

**Table 5:** NaCl-responsive transcripts related to signal transduction and hormone biosynthesis.

	number of genes					
family, group, or pathway	in genome	on array	signal above bkgnd.	> 2 fold up	> 2 fold down	assignment to STEM profiles (Fig. 2) cluster:# of genes

kinases	979	921	426	164	66	n,o:1;a:61;b:69;c:11;d:7; e:1;f:10;g:2;h:4;i:16;j:30; k:3;l:1;n:1;o:12;p:1
PPC:1 *	564	530	238	105	38	a:36;b:49;c:6;d:5;e:1; f:3;g:2;h:3;i:6;j:17;k:1; l:1;n:1;o:10
1.11.1 LLDK*	41	40	22	13	3	a:8;b:4;g:1;j:1;o:2
1.12.2 LRR II & X*	18	17	10	4	0	a:1;b:3
1.12.4 LRR XI & XII	45	44	27	8	5	a:2;b:2;c:1;d:2;f:1; h:1;i:1;j:3;n:1
1.2.2 RLCK VII*	45	44	25	19	0	a:6;b:9;c:1;d:2;e:1;f:1
1.5.1 WAK	27	23	11	6	5	b:3;c:1;j:3;l:1;o:1
1.6.2 PERK*	18	17	6	4	0	a:1;b:3
1.7.1 S-Domain (type 1)	14	14	10	7	0	a:3;b:3;c:1
1.7.2 DUF 26*	66	61	29	17	5	a:5;b:11;f:1;i:1;j:2;o:1
PPC:2	52	51	22	6	6	a:3;b:2;f:1;i:2;j:2;k: 1;o:1
PPC:4 *	279	265	133	47	18	n,o:1;a:20;b:18;c:5; d:1;f:5;i:6;j:10;o:1
4.1 MAP3K*	51	48	20	10	2	a:6;b:3;d:1;i:1
4.2 CRK*	123	119	58	21	10	n,o:1;a:5;b:10;c:3; f:3;i:3;j:4;o:1
4.2.6 RPS6K *	40	40	16	7	1	n,o:1;b:6;c:1;f:1
4.4 unclassified*	27	23	8	7	1	a:1;b:4;c:1;f:1;j:1
phosphatase*	125	116	64	24	9	a:9;b:11;c:3;d:1;g:1; i:5;j:1;k:1
PPC:6.1 (STKs)	23	19	12	0	3	i:2
PPC:6.2 DSP	8	8	6	1	2	a:1;i:1;j:1
PPC:6.3 PP2C *	64	62	38	22	3	a:7;b:11;c:3;g:1;i:1;k:1
						
response regulator: A *	11	11	8	4	1	a:1;b:1;c:2;i:1
similar to MLO proteins*	14	14	5	3	2	b:3;i:1;j:1
14-3-3 proteins*	13	13	8	0	5	i:1;j:4
						
ethylene biosynthesis	28	27	16	6	5	a:2;b:3;f:1;i:2;j:2
jasmonic acid biosynthesis*	20	19	11	7	3	a:1;b:5;d:1;j:2;o:1
IAA biosynthesis*	10	9	7	2	2	a:1;f:1;h:1;i:1;n:1
auxin transport*	8	8	4	3	1	a:2;b:1;i:1
Lateral Organ Boundaries class I*	36	34	11	7	0	a:2;b:1;c:3;g:1;p:1

Groups of genes that are significantly enriched in at least one STEM profile at a FDR of < 5% are marked with an asterisk (*). The final column details the frequency of assignment of individual genes to specific STEM profiles identified by corresponding letters in Figure 2. Functional categories in this table are defined according to the following sources: kinases and phosphatases [62]; hormone biosynthesis [23]; all others: [25, 37].

**Table 6:** Transcription factors.

	number of genes					
family, group, or pathway	in genome	on array	signal above bkgnd.	> 2 fold up	> 2 fold down	assignment to STEM profiles (Fig. 2) cluster:# of genes

ALFIN*	7	7	4	0	3	i:3
AP2/EREBP*	146	138	76	50	6	a:8;b:31;c:6;d:3;e:1; f:2;h:1;i:1;j:1;k:1;l:1; o:2
ARF*	23	20	12	4	0	a:3;d:1;f:1
AS2*	42	39	15	7	3	a:2;b:1;c:3;g:1;i:2;j:1; p:1
AUX/IAA	29	28	15	7	1	a:4;b:3;d:1;f:1;i:1;j:1
B3	39	36	8	2	2	a:1;b:1;i:2
bHLH	163	148	62	15	14	a:8;b:4;c:3;d:1;h:1;i:7; j:4;k:2;o:1;p:1
bZIP	75	68	42	11	11	a:5;b:5;c:2;i:3;j:4;l:1; o:2;p:1
C2C2-co-like*	31	31	13	5	2	b:1;d:1;e:1;f:2;i:1;j:1
C2C2-DOF*	36	31	15	8	1	b:5;c:1;d:2;g:1;k:1
C2C2-GATA*	29	27	14	3	3	b:3;d:2;h:1;i:1;j:1
C2H2*	130	122	57	22	14	a:5;b:15;c:1;d:1;f:1; i:5;j:6;o:2
C3H	33	31	13	5	2	a:1;b:3;c:1;j:1;n:1
CCAAT-HAP2	10	10	5	3	0	b:2;l:1
CCAAT-HAP3	11	11	7	2	2	a:2;i:1;j:1
CCAAT-HAP5	13	12	5	3	1	a:1;c:1;f:1;o:1
GARP-ARR-BARR19	12	12	4	1	1	b:1
GARP-G2-like*	43	43	26	8	8	b:4;c:1;d:3;f:1;i:3; j:4
GeBP	16	14	4	0	0	n/a
GRAS*	32	30	14	4	5	b:1;c:1;d:1;i:2;j:2;p:1
HB*	94	92	40	15	3	a:3;b:9;d:1;e:1;f:2;i:1; j:1;l:2;m:1
HMG	10	8	5	0	4	i:1;j:2;n:1
HSF*	24	23	13	10	1	a:6;b:4;d:1
JUMONJI	13	13	4	2	0	a:1;b:1;d:1
LIM	6	6	4	0	3	h:1;j:2;o:1
MADS	107	96	17	2	7	a:1;f:1;i:2;j:3;o:1
MYB*	203	188	84	33	12	a:9;b:18;c:1;d:5;f:2;i:3; j:5;n:1;o:1
NAC*	113	107	47	26	7	a:9;b:10;c:1;d:1;e:1;f:2; g:1;j:4;o:2
Nin-like	14	14	6	0	2	j:2
PHD	11	10	4	1	1	b:1;i:1
PLATZ*	9	8	5	2	0	b:2
SBP	16	15	8	3	1	a:1;b:2;i:1
TAZ	9	9	4	2	0	b:1;d:1;f:1
TCP	24	23	5	0	0	d:1
Trihelix	28	28	15	3	5	a:1;b:2;i:3;j:1;n:1
TUB	11	10	6	1	2	a:1;i:2
WRKY*	72	65	35	18	8	a:4;b:10;c:2;d:2;i:4; j:2;l:1
ZF-HD	15	15	6	1	2	d:1;j:2
ZIM*	15	12	10	8	1	b:3;c:3;d:1;f:1;o:1

Groups of genes that are significantly enriched in at least one STEM profile at a FDR of < 5% are marked with an asterisk (*). The final column details the frequency of assignment of individual genes to specific STEM profiles identified by corresponding letters in Figure 2. Functional categories in this table are defined according to the Database of Arabidopsis Transcription Factors [81]. The following small transcription factor families, which are each represented by probes for three or fewer genes on the microarray, are not listed in this table: ARID, BES1, C2C2-YABBY, CAMTA, CCAAT-DR1, CPP, E2F/DPE2FC, EIL, FHA, GIF, GRF, HRT-like, LFY, LUG, MBF1, NZZ, PBF-2-like(Whirly), PcG, S1Fa-like, SAP, SRS, ULT, VOZ.
